# Dietary Fructose Intake, Metabolic Profile, and Liver Disease-Related Quality of Life in Adults with Obesity and MASLD: A Cross-Sectional Study

**DOI:** 10.3390/metabo16100706

**Published:** 2026-09-24

**Authors:** Berna Güleç, Ayşe Betül Bilen, Dilek Baykal

**Affiliations:** 1 Department of Nutrition and Dietetics, Atlas University, Istanbul 34408, Türkiye; 232108030@st.atlas.edu.tr (B.G.); ayse.demirbas@atlas.edu.tr (A.B.B.); 2 Department of Nursing, Faculty of Health Sciences, Atlas University, Istanbul 34408, Türkiye

**Keywords:** metabolic dysfunction-associated steatotic liver disease, fructose intake, insulin resistance, obesity, quality of life, dietary assessment

## Abstract

**Highlights:**

**What are the main findings?**

- Questionnaire-derived fructose intake was modestly associated with waist circumference in the crude analysis, but this association was no longer significant after adjustment for total energy intake. Fructose intake was not associated with HOMA-IR or LDQOL.
- Male sex was associated with higher LDQOL scores, whereas lunch skipping and higher HbA1c were associated with lower LDQOL scores in the adjusted model.

**What are the implications of the main findings?**

- These findings highlight the importance of accounting for total energy intake when evaluating associations between dietary fructose and metabolic outcomes in adults with obesity and MASLD.
- The observed associations of meal pattern, glycaemic control, and sex with LDQOL warrant confirmation in prospective studies and should not be interpreted as evidence that modifying these factors will improve quality of life.

**Abstract:**

**Background/Objectives**: Metabolic dysfunction-associated steatotic liver disease (MASLD) is associated with obesity, insulin resistance, and dietary factors. This study investigated the associations of dietary fructose intake and insulin resistance with liver disease-related quality of life (LDQOL) and factors associated with LDQOL in adults with obesity and MASLD. **Methods**: This cross-sectional study included 169 adults with obesity (BMI ≥ 30 kg/m^2^) and MASLD. Dietary intake was assessed using a single 24 h recall, while habitual fructose exposure was estimated using a fructose consumption frequency questionnaire as the primary fructose variable. Insulin resistance and quality of life were assessed using HOMA-IR and LDQOL 1.0, respectively. A parsimonious multivariable linear regression model identified factors independently associated with LDQOL. **Results**: Questionnaire-derived fructose intake was modestly correlated with waist circumference (ρ = 0.26, *p* < 0.001), but this association was no longer significant after adjustment for total energy intake (partial ρ = 0.024, *p* = 0.761). Fructose intake was not associated with HOMA-IR or LDQOL. Mean HOMA-IR and LDQOL scores were 4.89 ± 3.81 and 50.94 ± 12.69, respectively. The multivariable model explained 27.7% of LDQOL variance (adjusted R^2^ = 0.236). Male sex was associated with higher LDQOL scores, whereas lunch skipping and higher HbA1c were associated with lower scores. **Conclusions**: The crude association between questionnaire-derived fructose intake and waist circumference was not retained after adjustment for total energy intake, and fructose intake was not associated with HOMA-IR or LDQOL. Associations of sex, lunch skipping, and glycaemic control with LDQOL warrant prospective confirmation and should not be interpreted as evidence that modifying these factors will improve quality of life.

## 1. Introduction

Metabolic dysfunction-associated steatotic liver disease (MASLD), formerly termed non-alcoholic fatty liver disease (NAFLD), is the most prevalent chronic liver disease worldwide and is defined by hepatic steatosis in the presence of at least one cardiometabolic risk factor and the absence of alternative causes of steatosis. Following the international Delphi consensus in 2023, the adoption of the MASLD nomenclature reflects a greater emphasis on the metabolic basis of the disease and provides a clinically relevant framework for diagnosis and management [1,2].

MASLD encompasses a broad spectrum ranging from simple steatosis to metabolic dysfunction-associated steatohepatitis (MASH), progressive fibrosis, cirrhosis, hepatocellular carcinoma, and liver-related mortality [2]. In addition to hepatic complications, MASLD is strongly associated with obesity, type 2 diabetes mellitus (T2DM), cardiovascular disease, and chronic kidney disease, making it a multisystem metabolic disorder with substantial global health and economic consequences [3,4].

Recent estimates indicate that approximately one-third of the adult population worldwide is affected by MASLD, with prevalence continuing to increase in parallel with the global epidemics of obesity and T2DM [5].

Insulin resistance (IR) is widely recognized as a central mechanism underlying the development and progression of MASLD. Impaired insulin signalling promotes increased adipose tissue lipolysis, resulting in excessive delivery of free fatty acids to the liver and enhanced hepatic triglyceride accumulation. Simultaneously, hepatic insulin resistance fails to suppress de novo lipogenesis while maintaining lipogenic signalling, thereby exacerbating hepatic steatosis. These metabolic alterations contribute to dyslipidaemia, chronic low-grade inflammation, oxidative stress, mitochondrial dysfunction, and progressive liver injury, ultimately increasing the risk of both hepatic and extrahepatic complications [6,7].

Among modifiable lifestyle factors, dietary habits play a pivotal role in the pathogenesis of MASLD. Particular attention has focused on fructose because of its unique hepatic metabolism and its capacity to stimulate de novo lipogenesis independently of insulin regulation. Excessive fructose intake, particularly from sugar-sweetened beverages and ultra-processed foods, has been associated with increased visceral adiposity, hepatic fat accumulation, oxidative stress, uric acid production, intestinal barrier dysfunction, and alterations in the gut microbiota, all of which may contribute to metabolic dysfunction and liver disease progression [8,9]. Nevertheless, recent evidence suggests that the adverse metabolic effects of fructose depend not only on the amount consumed but also on total energy intake, dietary source, overall dietary pattern, and the presence of obesity and other metabolic risk factors. Consequently, whether dietary fructose independently contributes to insulin resistance and MASLD severity remains a matter of ongoing debate [10].

Beyond its hepatic and metabolic consequences, MASLD is increasingly recognized as a disease that substantially impairs patient-reported outcomes and health-related quality of life (HRQOL). Individuals with MASLD frequently experience fatigue, reduced physical functioning, impaired work productivity, depression, anxiety, and diminished social well-being, even in the absence of advanced fibrosis or cirrhosis [11]. Disease-specific quality of life appears to be influenced by multiple interacting factors, including obesity, glycemic control, metabolic comorbidities, lifestyle behaviours, and liver disease severity. Sex may also influence health-related quality of life among individuals with MASLD. Recent evidence suggests that female sex is associated with poorer health-related quality of life in patients with MASLD/NAFLD. In a cross-cultural study of patients with NAFLD from Spain and the United Kingdom, female sex was a significant predictor of poorer quality of life in both cohorts, with female participants showing worse emotional function, greater fatigue, and higher body mass index, which were associated with lower quality of life [12]. Similarly, a recent cross-sectional study from China identified sex as an independent factor associated with health-related quality-of-life in patients with NAFLD [13]. These findings are consistent with broader evidence indicating clinically relevant sex-related differences in the epidemiology, pathophysiology, and clinical manifestations of MASLD [14]. Therefore, sex may represent an important demographic factor when evaluating factors associated with quality of life in MASLD. Given the multifactorial nature of quality of life in MASLD, demographic characteristics may interact with metabolic and lifestyle-related factors to influence patient-reported outcomes. Among these modifiable factors, dietary intake—and particularly fructose consumption—has received increasing attention because of its potential links with metabolic dysfunction and liver disease progression. However, the extent to which dietary factors, particularly fructose consumption, contribute to quality of life remains poorly understood.

Although accumulating evidence has linked excessive fructose intake with obesity, insulin resistance, and hepatic steatosis, few studies have simultaneously evaluated the relationships among dietary fructose intake, insulin resistance, and liver disease-specific quality of life in adults with obesity and MASLD.

Clarifying these interrelationships may improve understanding of the nutritional and metabolic correlates of patient-reported outcomes. Therefore, the present study aimed to investigate the associations between dietary fructose intake, insulin resistance, and liver disease-specific quality of life (LDQOL) in adults with obesity and MASLD and to identify demographic, clinical, metabolic, and dietary factors independently associated with LDQOL.

## 2. Materials and Methods

### 2.1. Study Design and Participants

This single-center, cross-sectional study was conducted between March and June 2025 at the Nutrition and Diet Outpatient Clinic of one State Hospital. For the revised multivariable regression model including nine predictors, the required sample size was estimated based on a medium effect size (f^2^ = 0.15), α = 0.05, and 80% statistical power. Under these assumptions, a minimum of approximately 114 participants was required. The available sample of 169 participants therefore exceeded this requirement.

Hepatic steatosis and ultrasonographic steatosis grade were identified from routine clinical ultrasonography records. Based on the documented ultrasonographic findings, participants were classified into three steatosis grades (Grade 1, Grade 2, and Grade 3).

Adults aged 18–65 years with obesity (BMI ≥ 30 kg/m^2^) and ultrasonographically confirmed MASLD were eligible for inclusion. Participants reporting alcohol consumption, viral hepatitis, other chronic liver diseases, pregnancy or lactation, or severe systemic diseases were excluded. The study was conducted in accordance with the ethical principles of the Declaration of Helsinki.

The study protocol was approved by the Clinical Research Ethics Committee of Istanbul Atlas University (protocol code 2025/01-35, dated 20 January 2025). Written informed consent was obtained from all participants before enrollment.

Detailed screening and recruitment logs were not systematically maintained; therefore, the total number of individuals assessed for eligibility, the number excluded before enrollment, the number who declined participation, and the number omitted because of incomplete records could not be determined retrospectively. In addition, recruitment was not prospectively documented as consecutive recruitment, and therefore we cannot confirm that all eligible patients presenting to the clinic during the study period were recruited consecutively.

### 2.2. Data Collection and Study Instruments

Data were collected using a structured Patient Information Form, a 24 h Dietary Recall Form, the Liver Disease Quality of Life Instrument 1.0 (LDQOL 1.0), and a Fructose Consumption Frequency Questionnaire.

#### 2.2.1. Patient Information Form

The data collection form was developed by researchers based on the relevant literature and previously published studies [1,2,3,4,5,6,7,8,9,10,11]. The form collected information on participants’ sociodemographic characteristics, anthropometric measurements, biochemical parameters, and dietary habits. Whether they currently smoke, their lunch consumption pattern, and their use of nutritional supplements (vitamins, minerals, etc.), among other factors, were assessed. Information on previous nutritional counseling, prescribed diets, recent weight-loss attempts, changes in dietary following MASLD diagnosis, and time since MASLD diagnosis was not systematically collected and was therefore unavailable for inclusion in the statistical analyses. Although body weight was measured at the study visit, systematic weight-history data, including the magnitude and timing of actual recent weight loss or weight gain, were not available.

No additional laboratory or radiological examinations were performed specifically for the study. All biochemical measurements and ultrasonographic findings, including steatosis grade, were retrieved from participants’ medical records as part of routine clinical care. The ultrasonographic steatosis grade documented in the medical records was used without independent re-grading by the investigators. Alcohol consumption was assessed by self-report as a binary variable (yes/no), and participants reporting alcohol consumption were excluded. Quantitative alcohol intake was not assessed. Participants with known chronic liver diseases or other recognized secondary causes of hepatic steatosis were excluded according to the study eligibility criteria. Fibrosis-specific assessments, including FIB-4 or elastography, were not available; therefore, ultrasonographic steatosis grade was interpreted specifically as the degree of hepatic steatosis rather than as a measure of fibrosis stage or overall MASLD severity. Body weight, height, and waist circumference were measured using standardized procedures. Anthropometric measurements were performed by the same trained dietitian according to standardized procedures. BMI and waist-to-height ratios were subsequently calculated.

#### 2.2.2. 24 H Dietary Recall and Fructose Consumption Frequency Questionnaire

Dietary intake was assessed using a single 24 h dietary recall obtained through face-to-face interviews conducted by a trained dietitian at the same study visit as the other questionnaires. Participants were asked to report all foods and beverages consumed during the preceding day. Accordingly, recalls collected on Mondays reflected Sunday intake, whereas recalls collected from Tuesday through Friday reflected weekday intake. Thus, both weekday and weekend days were represented across the study sample; however, only one recall was obtained per participant and repeated recalls were not performed. Standard household measures and food portion-size photographs were used to improve the accuracy of portion-size estimation. Daily energy, macronutrient, micronutrient, glucose, and fructose intakes were calculated using the Nutrition Information System software (BeBiS version 9.0; BebisPro for Windows, Stuttgart, Germany), which incorporates nutrient composition data for foods commonly consumed in Türkiye. The fructose estimate derived from the single 24 h dietary recall was considered a short-term dietary estimate and was used primarily to characterize dietary intake. Habitual fructose exposure was assessed using a fructose consumption frequency questionnaire developed based on previously published instruments [15,16]. The questionnaire assessed the habitual frequency and portion sizes of fructose-containing foods and beverages, including naturally occurring fructose sources (e.g., fruits, vegetables, and honey) and processed foods and sugar-containing beverages [17]. Fructose intake (g/day) was estimated from the reported consumption frequency and portion size, and this questionnaire-derived estimate was used as the primary fructose exposure variable in the statistical analyses. Free and added fructose were not separately quantified as distinct exposure variables. Detailed dietary intake data derived from the 24 h recall are provided in Supplementary Table S1.

#### 2.2.3. Liver Disease Quality of Life Instrument 1.0 (LDQOL 1.0)

Health-related quality of life was assessed using the Liver Disease Quality of Life Instrument (LDQOL 1.0), originally developed by Gralnek et al. [18] for patients with chronic liver disease. The Turkish version of the instrument was validated by Sarper [19]. LDQOL 1.0 is a disease-specific instrument designed to evaluate the impact of chronic liver disease on health-related quality of life. The disease-specific domains assessed liver disease-related symptoms, effects of liver disease on daily activities, concentration, memory, social interaction, health distress, sleep, loneliness, hopelessness, perception of liver disease, sexual functioning, and sexual problems. Domain scores were transformed to a 0–100 scale according to the LDQOL scoring procedure, with higher scores indicating better health-related quality of life. For the present study, the overall LDQOL score was calculated as the arithmetic mean of the available domain scores. When one or more domain scores were unavailable, the overall LDQOL score was calculated from the remaining available domain scores. No participants had missing overall LDQOL scores. In the present sample, the LDQOL 1.0 demonstrated good internal consistency among participants with complete data across all 12 disease-specific domains (Cronbach’s α = 0.860).

### 2.3. Biochemical Parameters and Assessment of Insulin Resistance

Venous blood samples were collected after an overnight fast. Serum triglycerides (TG), low-density lipoprotein cholesterol (LDL-C), high-density lipoprotein cholesterol (HDL-C), alanine aminotransferase (ALT), aspartate aminotransferase (AST), fasting plasma glucose, fasting insulin, and glycated haemoglobin (HbA1c) concentrations were obtained from routine laboratory records. The questionnaires, dietary assessments, anthropometric measurements and quality-of-life assessment were performed during the participants’ clinical evaluation period. Routine laboratory tests and ultrasonographic findings were obtained from the medical records corresponding to this clinical evaluation. However, the exact dates of the routine laboratory and ultrasonographic assessments and the study-specific assessments were not systematically recorded; therefore, a reliable maximum interval between these assessments could not be determined.

Insulin resistance was estimated using the homeostatic model assessment of insulin resistance (HOMA-IR), calculated as follows:

HOMA-IR = fasting glucose (mg/dL) × fasting insulin (µIU/mL)/405

### 2.4. Statistical Analysis

Statistical analyses were performed using IBM SPSS Statistics for Windows (version 29.0; IBM Corp., Armonk, NY, USA). The normality of continuous variables was assessed using the Shapiro–Wilk test together with visual inspection of histograms and Q–Q plots. Continuous variables are presented as mean ± standard deviation (SD) or median (interquartile range, IQR), according to data distribution, whereas categorical variables are presented as frequencies and percentages.

Comparisons between two independent groups were performed using the independent-samples *t*-test or the Mann–Whitney *U* test, as appropriate. Comparisons among MASLD steatosis grades were performed using one-way analysis of variance (ANOVA) or the Kruskal–Wallis test, as appropriate. When significant differences were identified, Dunn–Bonferroni post hoc tests were applied for pairwise comparisons. Effect sizes for Kruskal–Wallis analyses were calculated using epsilon-squared (ε^2^).

Associations among anthropometric, metabolic, dietary, insulin resistance, and liver disease-related quality-of-life variables were assessed using Spearman’s rank correlation coefficients and visualized with a correlation heatmap. Given the strong correlation between questionnaire-derived fructose intake and total energy intake, additional energy-adjusted analyses were performed using partial Spearman correlation coefficients controlling for total energy intake. These analyses examined the associations of questionnaire-derived fructose intake with waist circumference, HOMA-IR, and LDQOL total score.

A parsimonious multiple linear regression model was constructed to identify independent factors associated with LDQOL total score. Covariates were selected based on their biological and clinical relevance to quality of life and the primary objectives of the study, while limiting model complexity relative to the available sample size. The model included age, sex, dietary supplement use, lunch skipping, HbA1c, HOMA-IR, questionnaire-derived fructose intake, total energy intake, and ultrasonographic steatosis grade. For categorical variables, female sex and dietary supplement use were used as the reference categories. Lunch skipping was dichotomized as no lunch consumption versus all other lunch-consumption categories, with the latter used as the reference category. Ultrasonographic steatosis grade was entered as an ordinal variable (Grades 1–3). Total energy intake was included to account for potential confounding of the association with fructose intake. Regression assumptions, including linearity, normality of residuals, homoscedasticity, independence of errors, and multicollinearity, were evaluated. Variance inflation factors were examined to assess multicollinearity.

As a sensitivity analysis, the plausibility of reported energy intake was evaluated using the ratio of reported energy intake to estimated basal metabolic rate (EI/BMR), according to the Goldberg/Black approach [20]. BMR was estimated using the sex- and age-specific Schofield equations [21]. For the single-day dietary assessment (d = 1), EI/BMR values between 0.87 and 2.75 were considered plausible, assuming a physical activity level (PAL) of 1.55. Participants with EI/BMR values outside these limits were considered potential implausible energy reporters. The primary correlation analyses between questionnaire-derived fructose intake and waist circumference, HOMA-IR, and LDQOL total score were repeated after excluding these participants. Because individual physical activity levels were not available, the assumed PAL of 1.55 was used only for this sensitivity analysis.

A two-sided *p*-value < 0.05 was considered statistically significant.

## 3. Results

### 3.1. Characteristics of Participants

A total of 169 individuals with MASLD were included in this cross-sectional study. The mean age of the participants was 45.94 ± 11.44 years. The majority of participants were female (65.1%) and had graduated from high school (39.6%). Regarding lifestyle and clinical characteristics, 31.4% of participants were current smokers, and 16.6% reported using dietary supplements.

According to hepatic steatosis grading, 52.1% of participants were classified as Grade 1, 37.3% as Grade 2, and 10.7% as Grade 3. Regular sugar consumption was reported by 94.1% of participants, whereas 30.8% reported no lunch consumption. Baseline demographic, lifestyle, and clinical characteristics of the participants are presented in Table 1.

### 3.2. Anthropometric, Metabolic, Dietary, and Quality of Life Characteristics of Patients

Participants had a mean BMI of 35.62 ± 3.97 kg/m^2^, and a mean waist-to-height ratio of 0.673 ± 0.063. The mean systolic and diastolic blood pressures were 111.18 ± 15.21 mmHg and 81.57 ± 9.63 mmHg, respectively. The mean HOMA-IR value was 4.89 ± 3.81, indicating a high degree of insulin resistance within the study population. Mean fasting plasma glucose, HbA1c, triglyceride, LDL-C, ALT, and AST levels were 126.92 ± 56.88 mg/dL, 6.64 ± 1.82%, 231.76 ± 178.99 mg/dL, 143.13 ± 53.72 mg/dL, 65.62 ± 71.67 U/L, and 44.98 ± 78.10 U/L, respectively.

Dietary assessment showed a mean daily energy intake of 4620 ± 1267 kcal/day and a mean 24 h recall-derived fructose intake of 108.07 ± 32.72 g/day. The mean quality-of-life score was 50.94 ± 12.69. Detailed anthropometric, metabolic, dietary, and quality-of-life characteristics are presented in Table 2.

### 3.3. Correlations Between Dietary Intake, Metabolic Parameters, Insulin Resistance, and LDQOL

Spearman correlation analysis was performed to examine the relationships among dietary, anthropometric, metabolic, and quality-of-life variables (Figure 1). Questionnaire-derived fructose intake was positively correlated with waist circumference (ρ = 0.26, *p* < 0.001), total energy intake (ρ = 0.67, *p* < 0.001), and protein intake (ρ = 0.42, *p* < 0.001), but showed no significant correlation with HOMA-IR or LDQOL. HOMA-IR was positively correlated with HbA1c (ρ = 0.67, *p* < 0.001) and triglyceride levels (ρ = 0.24, *p* < 0.05). LDQOL was negatively correlated with age (ρ = −0.26, *p* < 0.01) and HbA1c (ρ = −0.24, *p* < 0.05), whereas positive correlations were observed with total energy intake (ρ = 0.26, *p* < 0.05) and protein intake (ρ = 0.31, *p* < 0.01).

Given the strong correlation between questionnaire-derived fructose intake and total energy intake (ρ = 0.669, *p* < 0.001), additional analyses controlling for total energy intake were performed. After energy adjustment, the association between questionnaire-derived fructose intake and waist circumference was attenuated and was no longer statistically significant (partial ρ = 0.024, *p* = 0.761). No significant energy-adjusted associations were observed with HOMA-IR (partial ρ = −0.102, *p* = 0.188) or LDQOL total score (partial ρ = −0.073, *p* = 0.345) (Table S3).

In the sensitivity analysis of energy-reporting plausibility, 103 of 169 participants (60.9%) were classified as plausible energy reporters, whereas 66 (39.1%) were classified as potential over-reporters; no participant was classified as a potential under-reporter. After exclusion of potential over-reporters, the positive association between questionnaire-derived fructose intake and waist circumference remained significant (ρ = 0.411, *p* < 0.001). Questionnaire-derived fructose intake remained unassociated with HOMA-IR (ρ = −0.172, *p* = 0.082) and LDQOL total score (ρ = 0.104, *p* = 0.294). Overall, the pattern of associations was consistent with the primary analysis (Table S2).

### 3.4. Factors Associated with Quality of Life: Multivariable Linear Regression Analysis

In the parsimonious multivariable linear regression model, the included covariates collectively explained 27.7% of the variance in LDQOL total score (R^2^ = 0.277; adjusted R^2^ = 0.236; F (9, 159) = 6.765, *p* < 0.001). Male sex, dietary supplement non-use, lunch skipping, and higher HbA1c were independently associated with LDQOL total score, whereas age, HOMA-IR, questionnaire-derived fructose intake, total energy intake, and ultrasonographic steatosis grade were not statistically significant in the adjusted model. Male sex was independently associated with a higher LDQOL score compared with female sex (B = 8.723, 95% CI: 4.737 to 12.709, *p* < 0.001). Notably, questionnaire-derived fructose intake was not independently associated with LDQOL after adjustment for total energy intake and the other covariates (B = −0.017, *p* = 0.615). Multicollinearity was low, with all variance inflation factors below 2.5 (Table 3).

## 4. Discussion

This cross-sectional study investigated the relationships among dietary fructose intake, insulin resistance, and disease-specific quality of life in adults with obesity and MASLD. Questionnaire-derived fructose intake showed a modest crude association with waist circumference, but this association was no longer statistically significant after adjustment for total energy intake. Fructose intake was not associated with HOMA-IR or LDQOL, and HOMA-IR was not independently associated with LDQOL. In the adjusted model, male sex was associated with higher LDQOL scores, whereas lunch skipping and higher HbA1c were associated with lower scores. These findings underscore the importance of accounting for total energy intake when interpreting fructose-related associations and indicate that the observed relationships with LDQOL are multifactorial.

### 4.1. Anthropometric and Metabolic Characteristics

The study population was characterized by severe obesity, pronounced central adiposity, and substantial metabolic dysfunction, all of which are hallmarks of MASLD. Participants had a mean BMI of 35.62 ± 3.97 kg/m^2^, and a mean waist-to-height ratio of 0.673 ± 0.063, indicating a markedly increased cardiometabolic risk. Although more than 89% of participants had Grade 1 or Grade 2 steatosis, the cohort demonstrated considerable metabolic impairment, including elevated HOMA-IR, HbA1c, triglyceride, and liver enzyme levels. These findings are consistent with evidence indicating that substantial metabolic dysfunction may be present even in individuals with lower ultrasonographic steatosis grades [22,23,24].

The strong positive correlation between BMI and waist circumference observed in the present study further highlights the close relationship between overall and central adiposity in individuals with MASLD. Nevertheless, growing evidence suggests that visceral adiposity is more closely associated with hepatic steatosis, insulin resistance, systemic inflammation, and adverse cardiometabolic outcomes than BMI alone [25]. Visceral fat contributes to increased free fatty acid delivery to the liver, chronic low-grade inflammation, altered adipokine secretion, and hepatic insulin resistance, thereby promoting hepatic lipid accumulation and disease progression [26]. Consequently, anthropometric indicators of central obesity, such as waist circumference and WHtR, are increasingly recognized as clinically relevant markers for identifying individuals at high metabolic risk [27].

Consistent with the central role of insulin resistance in MASLD pathophysiology, HOMA-IR was strongly correlated with HbA1c and modestly correlated with triglyceride concentrations in our study. These findings are in line with current evidence indicating that impaired insulin signalling contributes not only to glucose dysregulation but also to increased hepatic de novo lipogenesis, dyslipidaemia, and progressive metabolic dysfunction [28].

Interestingly, despite the predominance of Grade 1 and Grade 2 steatosis, participants exhibited marked metabolic abnormalities, including elevated HOMA-IR, HbA1c, and triglyceride levels. This finding suggests that the degree of metabolic impairment is not necessarily proportional to ultrasonographic steatosis grade. Rather, metabolic abnormalities in MASLD may reflect the complex interplay among adipose tissue dysfunction, hepatic insulin resistance, lipid overflow, and chronic low-grade inflammation [28,29].

Taken together, the anthropometric and metabolic profile of our cohort is representative of the typical obesity-related MASLD phenotype reported in contemporary clinical studies. Given that obesity, insulin resistance, and poor glycaemic control have been reported as important correlates of patient-reported outcomes in MASLD, this metabolic background provides an important context for interpreting the observed relationships between dietary fructose intake, insulin resistance, and disease-specific quality of life. Previous studies have shown that HRQOL in MASLD is influenced not only by liver disease severity but also by metabolic comorbidities, physical functioning, fatigue, and other obesity-related complications, highlighting the multifactorial nature of quality of life impairment in this population [30].

### 4.2. Nutrition and Liver Disease-Related Quality of Life

In the present study, long-term glycaemic control emerged as the metabolic parameter most consistently associated with liver disease-related quality of life, with higher HbA1c levels being associated with poorer LDQOL scores. This finding is consistent with previous studies demonstrating that poor glycaemic control is associated with poorer patient-reported outcomes through multiple mechanisms, including reduced physical functioning, increased fatigue, and a higher burden of diabetes-related symptoms [25]. Recent evidence also suggests that metabolic comorbidities, particularly type 2 diabetes and obesity, contribute substantially to impaired quality of life in individuals with MASLD, independent of liver disease severity.

Higher total energy and protein intakes were positively correlated with LDQOL scores in the crude analyses. Although participants reported a high mean daily energy intake, interpretation of these associations requires caution given the use of a single 24 h dietary recall and the presence of potential energy over-reporting in a substantial proportion of participants. Higher reported energy and protein intakes may also reflect differences in appetite, dietary intake patterns, or overall functional status rather than a direct beneficial effect of greater intake. Protein intake may additionally be relevant to nutritional adequacy and maintenance of muscle mass, which have been linked to physical functioning and health-related quality of life in chronic liver disease [31]. However, because of the cross-sectional design and the potential for dietary measurement error and residual confounding, these associations should not be interpreted causally.

### 4.3. Dietary Fructose Intake and Associated Factors

It should be noted that the fructose estimates in the present study represent total dietary fructose from both naturally occurring and processed food sources and should not be interpreted as free or added sugar intake. Accordingly, direct comparison of the observed fructose intake with recommendations for free sugar intake would not be appropriate. The fructose estimates observed in this cohort should therefore be interpreted as descriptive measures of total fructose exposure rather than as evidence of exceeding guideline-based free sugar thresholds.

In the crude correlation analysis, questionnaire-derived fructose intake was positively associated with waist circumference (ρ = 0.26, *p* < 0.001) but was not significantly associated with HOMA-IR (ρ = −0.10) or LDQOL (ρ = 0.12). However, because questionnaire-derived fructose intake was strongly correlated with total energy intake, additional energy-adjusted analyses were performed. After adjustment for total energy intake, the association between questionnaire-derived fructose intake and waist circumference was substantially attenuated and was no longer statistically significant. This finding suggests that the crude association between questionnaire-derived fructose intake and waist circumference was strongly influenced by total energy intake and did not persist after energy adjustment.

Previous studies have reported associations between high fructose intake, particularly from sugar-sweetened beverages and ultra-processed foods, and markers of central adiposity, including waist circumference and visceral fat accumulation [32,33]. However, the present findings indicate that the crude association between fructose intake and central adiposity may be substantially influenced by total energy intake, emphasizing the importance of considering overall energy consumption when interpreting fructose–adiposity relationships.

Questionnaire-derived fructose intake was not significantly associated with HOMA-IR, HbA1c, liver enzyme concentrations, or disease-specific quality of life. Similar findings have been reported in studies suggesting that the metabolic effects associated with fructose consumption may depend on total energy intake, dietary source, and the underlying metabolic status of the population rather than fructose intake alone [10]. In populations with established obesity and MASLD, where insulin resistance and metabolic dysfunction are already highly prevalent, the independent contribution of fructose intake to metabolic outcomes may therefore be difficult to distinguish [34]. Taken together, our findings do not support an independent association between questionnaire-derived fructose intake and central adiposity, insulin resistance, or disease-specific quality of life after accounting for total energy intake.

### 4.4. Factors Independently Associated with Liver Disease-Related Quality of Life

The parsimonious multivariable regression analysis showed that male sex, dietary supplement non-use, lunch skipping, and higher HbA1c were independently associated with LDQOL. In contrast, age, HOMA-IR, questionnaire-derived fructose intake, total energy intake, and ultrasonographic steatosis grade were not statistically significant in the adjusted model. Male participants had higher adjusted LDQOL scores than female participants, indicating poorer disease-specific quality of life among women in this cohort.

Beyond statistical significance, the magnitude of the regression coefficients should also be considered. After multivariable adjustment, male participants had LDQOL scores approximately 8.7 points higher than female participants (95% CI: 4.7 to 12.7), whereas lunch skipping was associated with an approximately 5.2-point lower LDQOL score (95% CI: −9.0 to −1.5). Each 1-percentage-point increase in HbA1c was associated with an approximately 1.9-point lower LDQOL score (95% CI: −3.4 to −0.5). Dietary supplement non-use was associated with an approximately 5.0-point higher LDQOL score, although the confidence interval was wide (95% CI: 0.1 to 9.8), indicating greater uncertainty regarding the magnitude of this association. To our knowledge, a well-established minimally important difference for the LDQOL 1.0 total composite score in adults with MASLD has not been established; therefore, these effect estimates should not be interpreted as definitively clinically meaningful solely on the basis of statistical significance.

Male sex was independently associated with higher LDQOL scores compared with female sex in the adjusted model. Previous studies have reported sex-related differences in health-related quality of life among individuals with NAFLD/MASLD, although findings vary across populations and clinical settings [12,13,35]. More recent evidence also highlights sex-related heterogeneity in the epidemiology, metabolic phenotype, and clinical characteristics of MASLD, potentially reflecting differences in hormonal status, adipose-tissue distribution, and other metabolic and lifestyle factors [14,36]. In the present study, the association persisted after adjustment for the measured demographic, clinical, metabolic, and dietary covariates. Nevertheless, residual confounding by unmeasured sex-specific factors, including menopausal status and psychosocial characteristics, cannot be excluded. Therefore, this finding should not be interpreted as evidence that male sex is inherently protective; rather, sex may represent a marker of heterogeneity in patient-reported outcomes. Further longitudinal and sex-stratified studies are warranted.

Lunch skipping was independently associated with lower LDQOL scores. Previous studies have linked irregular meal patterns with adverse metabolic characteristics, including impaired glucose metabolism and insulin resistance. However, the cross-sectional design of the present study does not allow determination of whether lunch skipping contributes to poorer quality of life or instead reflects other behavioral, socioeconomic, or health-related characteristics. Dietary supplement non-use was associated with an approximately 5-point higher adjusted LDQOL score compared with supplement use. However, this finding should be interpreted with particular caution. Only 16.6% of participants reported dietary supplement use, and detailed information regarding supplement type, indication, dose, and duration was not available. Therefore, the observed association should not be interpreted as evidence that avoiding dietary supplements improves quality of life. Supplement use may be particularly susceptible to indication bias, healthy-user effects, and residual confounding; for example, individuals with greater symptom burden, comorbidities, or specific nutritional concerns may have been more likely to use supplements. Accordingly, this observational finding should be considered hypothesis-generating rather than a basis for nutritional recommendations.

Among the biochemical variables included in the model, HbA1c remained independently associated with LDQOL, with higher HbA1c levels associated with lower LDQOL scores. This finding is consistent with recent evidence demonstrating that dysglycemia and type 2 diabetes substantially increase symptom burden and impair health-related quality of life in patients with MASLD. In contrast, HOMA-IR was not independently associated with LDQOL after multivariable adjustment, suggesting that insulin resistance alone may not adequately explain disease-specific quality of life when long-term glycemic control and other clinical factors are considered [37,38].

Overall, these findings suggest that glycaemic control and meal-pattern characteristics are associated with LDQOL in adults with obesity and MASLD. Because of the cross-sectional design, these associations should not be interpreted as evidence that modifying glycaemic control or meal patterns will improve quality of life and require confirmation in prospective studies.

### 4.5. Strengths and Limitations

The main strength of this study is the simultaneous assessment of habitual fructose exposure, insulin resistance, and liver disease-related quality of life within a homogeneous clinical sample of adults with obesity and confirmed MASLD. The use of the validated LDQOL 1.0 instrument, energy-adjusted analyses, sensitivity analyses for implausible energy reporting, and a parsimonious multivariable model with multicollinearity diagnostics represent additional methodological strengths. This study provides a comprehensive evaluation in a Turkish population of the relationships among dietary fructose intake, metabolic characteristics, insulin resistance, and liver disease-related quality of life in adults with MASLD.

However, several limitations should be acknowledged. First, the cross-sectional design precludes causal inference, and the observed associations should therefore be considered hypothesis-generating. Second, participants were recruited from a single center, which may limit the generalizability of the findings. Selection bias cannot be excluded because the study was conducted in a nutrition and diet outpatient clinic. Detailed screening and recruitment logs were not systematically maintained; therefore, the numbers of individuals screened, excluded before enrollment, declining participation, or omitted because of incomplete records could not be determined retrospectively. In addition, recruitment was not prospectively documented as consecutive, and we therefore cannot confirm that all eligible patients presenting to the clinic during the study period were recruited consecutively. Third, dietary assessment relied on self-reported methods. Short-term dietary intake was assessed using a single 24 h dietary recall and was therefore subject to recall bias, day-to-day variation, and measurement error. A substantial proportion of participants were classified as potential energy over-reporters using the Goldberg/Black approach; although sensitivity analyses restricted to plausible energy reporters generally supported the main findings, the dietary estimates should be interpreted cautiously. Furthermore, the fructose questionnaire quantified total dietary fructose and did not allow naturally occurring fructose to be distinguished from free or added fructose.

Residual confounding also cannot be excluded. Detailed information on diabetes duration, specific glucose-lowering medications, hypertension and cardiovascular comorbidities, physical activity, depression and anxiety, and other relevant clinical and socioeconomic factors was not systematically available and therefore could not be incorporated into the multivariable model. Similarly, information on previous nutritional counseling, prescribed diets, recent weight-loss attempts, and time since MASLD diagnosis was not systematically available. Although body weight was measured at the study visit, systematic weight-history data, including the magnitude and timing of actual recent weight loss or weight gain, were not available. Although a parsimonious model was used to limit model complexity, the sample size may still restrict model stability and the ability to account for additional confounders.

MASLD diagnosis and steatosis grading were based on routine ultrasonographic records, and quantitative alcohol intake and fibrosis-specific measures were unavailable. Routine ultrasonography is operator-dependent and does not provide reliable fibrosis staging. Therefore, ultrasonographic grades should be interpreted specifically as steatosis grades rather than as measures of fibrosis or overall MASLD severity.

The interpretation of HOMA-IR also warrants caution. HOMA_IR is a fasting surrogate derived from fasting glucose and insulin concentration and does not provide a comprehensive assessment of whole-body insulin sensitivity, particularly peripheral insulin sensitivity, nor does it directly characterize β-cell compensation. Accordingly, the HOMA-IR findings in the present study should be interpreted specifically as reflecting fasting insulin resistance rather than overall insulin sensitivity. Future studies using dynamic metabolic testing and calculation of the Disposition Index would provide a more comprehensive assessment of peripheral insulin sensitivity and β-cell function.

In addition, although the LDQOL 1.0 was originally developed for patients with advanced chronic liver disease, it was used in the present study in a predominantly Grade 1–2 ultrasonographic MASLD population; its applicability and sensitivity in individuals with predominantly lower ultrasonographic steatosis grades should therefore be considered. Dietary supplement use was self-reported and uncommon, and the absence of information on supplement type, indication, dose, and duration makes this finding particularly susceptible to indication bias, healthy-user effects, and residual confounding. Lunch skipping was also self-reported and was dichotomized for the regression analysis, which may have introduced misclassification and reduced information on meal-pattern variability. Finally, multiple statistical comparisons were performed, increasing the possibility of chance findings; secondary and exploratory associations should therefore be interpreted cautiously. Future prospective and interventional studies using repeated dietary assessments, more detailed characterization of fructose sources, dynamic metabolic testing with assessment of the Disposition Index, and fibrosis-specific measures are needed to confirm these findings.

## 5. Conclusions

In conclusion, questionnaire-derived fructose intake was modestly associated with waist circumference in the crude analysis, but this association was not retained after adjustment for total energy intake. Questionnaire-derived fructose intake was not associated with HOMA-IR or LDQOL.

Although HOMA-IR was associated with glycemic and lipid-related metabolic markers, it was not independently associated with LDQOL. Instead, male sex was associated with higher LDQOL scores, whereas lunch skipping and higher HbA1c were associated with lower LDQOL scores. Dietary supplement non-use was also associated with higher LDQOL scores; however, given the small proportion of supplement users and the potential for indication bias and residual confounding, this finding should be interpreted cautiously and should not be considered evidence of a benefit from avoiding dietary supplements. Age, HOMA-IR, questionnaire-derived fructose intake, total energy intake, and ultrasonographic steatosis grade were not independently associated with LDQOL in the adjusted model.

Overall, these cross-sectional findings should not be interpreted as evidence that reducing fructose intake, changing meal patterns, altering supplement use, or improving glycaemic control will improve metabolic health or quality of life. Prospective and interventional studies are needed to determine whether changes in these factors are followed by changes in metabolic and patient-reported outcomes in adults with MASLD.

## Figures and Tables

**Figure 1:**
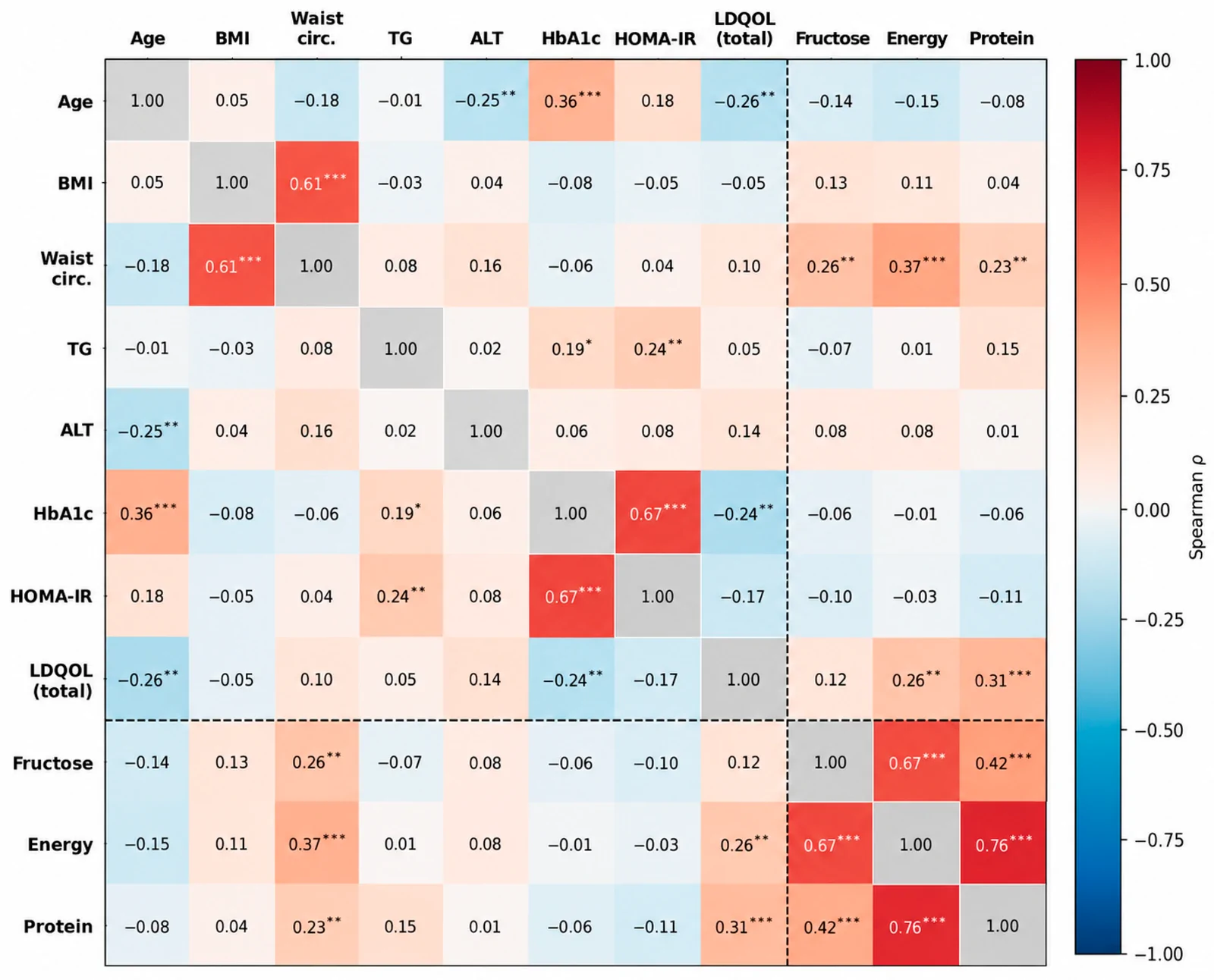
Spearman correlation matrix of clinical, metabolic, and dietary parameters in adults with obesity and MASLD (N = 169). Fructose, questionnaire-derived fructose intake. Colour intensity reflects the magnitude of the Spearman correlation coefficient (ρ); red indicates positive and blue indicates negative correlations. The dashed line separates clinical and metabolic parameters (upper-left block) from dietary parameters (lower-right block). Diagonal cells represent self-correlations (ρ = 1.00). Significance levels are indicated as follows: * *p* < 0.05; ** *p* < 0.01; *** *p* < 0.001. Abbreviations: BMI, body mass index; WC, waist circumference; TG, triglycerides; ALT, alanine aminotransferase; HbA1c, glycated haemoglobin; HOMA-IR, Homeostatic Model Assessment of Insulin Resistance; LDQOL, liver disease-related quality of life (total score).

**Table 1:** Characteristics of participants (*n* = 169).

Characteristics		Total (*n* = 169)
Age (M ± SD) (years)		45.94 ± 11.44
Sex, *n* (%)	Male	59 (34.9)
	Female	110 (65.1)
Marital status *n* (%)	Single	38 (22.5)
	Married	131 (77.5)
Education level, *n* (%)	Primary school	60 (35.5)
	Secondary school	18 (10.7)
	High school	67 (39.6)
	University or higher	24 (14.2)
Smoking, *n* (%)	Yes	53 (31.4)
	No	116 (68.6)
Alcohol use, *n* (%)	Yes	0 (0)
	No	169 (100)
Ultrasonographic steatosis grade, *n* (%)	Grade 1	88 (52.1)
	Grade 2	63 (37.3)
	Grade 3	18 (10.7)
Sugar use, *n* (%)	Yes	159 (94.1)
	No	10 (5.9)
Lunch consumption, *n* (%)	Regular	76 (45.0)
	Sometimes	40 (23.7)
	Mostly	1 (0.6)
	No	52 (30.8)
Supplement use, *n*(%)	Yes	28 (16.6)
	No	141 (83.4)

MASLD: Metabolic dysfunction-associated steatotic liver disease. Categorical data are presented as frequency (percentage).

**Table 2:** Anthropometric, metabolic, dietary, and quality-of-life characteristics of patients.

Variables	M ± SD	Median (Q1–Q3)
Anthropometric parameters		
BMI (kg/m^2^)	35.62 ± 3.97	34.89 (32.94–37.89)
Waist-to-height ratio	0.673 ± 0.063	0.664 (0.635–0.710)
Metabolic parameters		
Fasting glucose (mg/dL)	126.92 ± 56.88	110 (95–138)
Insulin (μIU/mL)	14.66 ± 5.67	13.90 (10.9–18)
HOMA-IR	4.89 ± 3.81	4.00 (2.70–5.50)
HbA1c (%)	6.64 ± 1.82	6.10 (5.7–6.9)
Triglycerides (mg/dL)	231.76 ± 178.99	198 (138–255)
LDL-C (mg/dL)	143.13 ± 53.72	141 (105–172)
HDL-C (mg/dL)	44.78 ± 11.56	44 (38–51)
ALT (U/L)	65.62 ± 71.67	50 (38–75)
AST (U/L)	44.98 ± 78.10	34 (25–45)
Systolic pressure (mmHg)	111.18 ± 15.21	112 (98–120)
Diastolic pressure (mmHg)	81.57 ± 9.63	80 (76–88)
Dietary intake		
Energy intake (kcal/day)	4620 ± 1267	4566 (3707–5509)
Carbohydrate (g/day)	649.28 ± 193.54	635.33 (524.29–766.26)
Protein (g/day)	118.08 ± 35.14	111.95 (96.79–139.39)
Fat (g/day)	160.68 ± 60.07	163.44 (120.19–196.07)
24 h recall-derived fructose intake (g/day)	108.07 ± 32.72	110 (92.54–127.38)
Quality of life		
LDQOL	50.94 ± 12.69	51.44 (39.13–61.93)

**Table 3:** Multivariable Linear Regression Analysis of Factors Associated with LDQOL Total Score.

Predictor	B	SE	Standardized β	95% CI	*p*	VIF
Age	−0.160	0.085	−0.145	−0.329 to 0.008	0.062	1.30
Sex (male) *	8.723	2.018	0.329	4.737 to 12.709	<0.001	1.27
Dietary supplement non-use	4.957	2.462	0.146	0.095 to 9.818	0.046	1.15
Lunch skipping	−5.249	1.878	−0.191	−8.958 to −1.541	0.006	1.03
HbA1c (%)	−1.945	0.721	−0.279	−3.370 to −0.521	0.008	2.36
HOMA-IR	0.495	0.341	0.148	−0.179 to 1.169	0.149	2.30
Questionnaire-derived fructose intake (g/day)	−0.017	0.033	−0.048	−0.081 to 0.048	0.615	1.99
Total energy intake (kcal/day)	0.001	0.001	0.136	−0.001 to 0.003	0.166	2.10
Ultrasonographic steatosis grade	−1.564	1.310	−0.083	−4.151 to 1.023	0.234	1.07
Model statistics: *n* = 169; R^2^ = 0.277; adjusted R^2^ = 0.236; F (9, 159) = 6.765; *p* < 0.001.						

* Female sex and dietary supplement use were used as the reference categories. The coefficient for dietary supplement non-use represents non-use versus use. Lunch skipping represents no lunch consumption versus all other lunch-consumption categories, with the latter used as the reference category. Ultrasonographic steatosis grade was entered as an ordinal variable (Grades 1–3). B, unstandardized regression coefficient; SE, standard error; β, standardized regression coefficient; CI, confidence interval; VIF, variance inflation factor; HbA1c, glycated hemoglobin; HOMA-IR, Homeostatic Model Assessment of Insulin Resistance; LDQOL, liver disease-related quality of life.

## Data Availability

The data presented in this study are available on request from the corresponding author. The data are not publicly available due to privacy restrictions pertaining to patient information.

## Supplementary Materials

The following supporting information can be downloaded at: https://www.mdpi.com/article/10.3390/metabo16100706/s1, Table S1: Daily nutrient intake by MASLD steatosis grade—Kruskal–Wallis test (N = 169); Table S2: Sensitivity analysis after excluding potential energy over-reporters; Table S3: Energy-adjusted associations between questionnaire-derived fructose intake and selected outcomes.

## Abbreviations

ALT	Alanine aminotransferase
AST	Aspartate aminotransferase
BMI	Body mass index
DNL	De novo lipogenesis
HbA1c	Glycated haemoglobin
HDL-C	High-density lipoprotein cholesterol
HOMA-IR	Homeostatic Model Assessment of Insulin Resistance
IQR	Interquartile range
LDL-C	Low-density lipoprotein cholesterol
LDQOL	Liver Disease Quality of Life
NAFLD	Non-alcoholic fatty liver disease
MASLD	Metabolic dysfunction-associated steatotic liver disease
SD	Standard deviation
TG	Triglycerides
VLDL-C	Very low-density lipoprotein
WC	Waist circumference

## References

[1] Rinella M.E., Lazarus J.V., Ratziu V., Francque S.M., Sanyal A.J., Kanwal F., Romero D., Abdelmalek M.F., Anstee Q.M., Arab J.P. (2024). A multisociety Delphi consensus statement on new fatty liver disease nomenclature. Ann. Hepatol..

[2] Sberna A.L., Bouillet B., Rouland A., Brindisi M.C., Nguyen A., Mouillot T., Duvillard L., Denimal D., Loffroy R., Vergès B. (2018). European Association for the Study of the Liver (EASL), European Association for the Study of Diabetes (EASD) and European Association for the Study of Obesity (EASO) clinical practice recommendations for the management of non-alcoholic fatty liver disease: Evaluation of their application in people with Type 2 diabetes. Diabet. Med..

[3] Byrne C.D., Armandi A., Pellegrinelli V., Vidal-Puig A., Bugianesi E. (2025). Μetabolic dysfunction-associated steatotic liver disease: A condition of heterogeneous metabolic risk factors, mechanisms and comorbidities requiring holistic treatment. Nat. Rev. Gastroenterol. Hepatol..

[4] Grocic M., Nguyen M., Sanyal A.J. (2026). Current trends in the epidemiology of obesity and the association between obesity and metabolic liver disease (MASLD/MASH). Diabetes Obes. Metab..

[5] Huang D.Q., Wong V.W., Rinella M.E., Boursier J., Lazarus J.V., Yki-Järvinen H., Loomba R. (2025). Metabolic dysfunction-associated steatotic liver disease in adults. Nat. Rev. Dis. Primers.

[6] (2015). Guideline: Sugars Intake for Adults and Children. https://www.who.int/publications/i/item/9789241549028.

[7] Rinella M.E., Neuschwander-Tetri B.A., Siddiqui M.S., Abdelmalek M.F., Caldwell S., Barb D., Kleiner D.E., Loomba R. (2023). AASLD Practice Guidance on the clinical assessment and management of nonalcoholic fatty liver disease. Hepatology.

[8] Younossi Z.M., Zelber-Sagi S., Lazarus J.V., Wong V.W.S., Yilmaz Y., Duseja A., Eguchi Y., Castera L., Pessoa M.G., Oliveira C.P. (2025). Global consensus recommendations for metabolic dysfunction-associated steatotic liver disease and steatohepatitis. Gastroenterology.

[9] Heyens L.J.M., Van Steenkiste C., Struyve M., Francque S.M., Koek G.H., Robaeys G., Kockaerts Y., Lowyck I., Stinkens K., MASLD Research Group (2026). MASLD Is Associated with Decreased General Health Perception, Quality of Life and Work Productivity in a Low Fibrosis Population. Liver Int..

[10] Jafari A., Faghfouri A.H., Nikpayam O. (2024). The effect of low-fructose diet on anthropometric and metabolic factors: A systematic review and meta-analysis. Nutr. Metab. Cardiovasc. Dis..

[11] Shea S., Lionis C., Kite C., Lagojda L., Uthman O.A., Dallaway A., Atkinson L., Chaggar S.S., Randeva H.S., Kyrou I. (2024). Non-alcoholic fatty liver disease and coexisting depression, anxiety and/or stress in adults: A systematic review and meta-analysis. Front. Endocrinol..

[12] Funuyet-Salas J., Martín-Rodríguez A., Pérez-San-Gregorio M.Á., Vale L., Robinson T., Anstee Q.M., Romero-Gómez M. (2024). Health-related quality of life in non-alcoholic fatty liver disease: A cross-cultural study between Spain and the United Kingdom. PLoS ONE.

[13] Lv K., Zhou R., Gu Y., Kong T., Chen Y., Shao Y., Shi J., Zhang W. (2025). Status and factors influencing health-related quality of life in patients with non-alcoholic fatty liver disease in Hangzhou: A cross-sectional study. BMJ Open.

[14] Kaushal K., Singh S., Anand A., Ramakrishna G., Kale P., Sharma R., Rewari B., Sarin S.K. (2026). Sex Differences in Metabolic Dysfunction-Associated Steatotic Liver Disease (MASLD): Epidemiology, Pathophysiology, and Clinical Implications. J. Clin. Exp. Hepatol..

[15] Kuyumcu A., Pürnak T., Yıldız E.A. (2019). Evaluation of fructose consumption in individuals with non-alcoholic fatty liver disease. Turk. J. Clin. Lab..

[16] Tengilimoğlu Metin M., Melekoğlu E. (2021). The relationship between stress status and consumption of fructose and processed food in university students. Çukurova J. Agric. Food Sci..

[17] Yararbaş Ç. (2023). The Relationship Between Dietary Fructose Intake and Metabolic Syndrome Parameters in Adults Admitted to the Internal Medicine Outpatient Clinic of a Private Hospital. Master’s Thesis.

[18] Gralnek I.M., Hays R.D., Kilbourne A., Rosen H.R., Keeffe E.B., Artinian L., Kim S., Lazarovici D., Jensen D.M., Busuttil R.W. (2000). Development and evaluation of the Liver Disease Quality of Life instrument in persons with advanced, chronic liver disease--the LDQOL 1.0. Am. J. Gastroenterol..

[19] Sarper B. (2007). Validity and Reliability of the Liver Disease Quality of Life Scale 1.0 for the Turkish population. Master’s Thesis.

[20] Black A.E. (2000). Critical evaluation of energy intake using the Goldberg cut-off for energy intake: Basal metabolic rate. A practical guide to its calculation, use and limitations. Int. J. Obes. Relat. Metab. Disord..

[21] Schofield W. (1985). Predicting basal metabolic rate, new standards and review of previous work. Hum. Nutr. Clin. Nutr..

[22] Do A., Zahrawi F., Mehal W.Z. (2025). Therapeutic landscape of metabolic dysfunction-associated steatohepatitis (MASH). Nat. Rev. Drug Discov..

[23] Wong R.J. (2024). Epidemiology of metabolic dysfunction-associated steatotic liver disease (MASLD) and alcohol-related liver disease (ALD). Metab. Target Organ Damage.

[24] Horn P., Tacke F. (2025). Key takeaways from the updated multidisciplinary European MASLD guidelines. eGastroenterology.

[25] Potter A.W., Friedl K.E. (2025). Cross-sectional analysis of visceral adipose tissue associations with obesity-related disease. Am. J. Clin. Nutr..

[26] Romanos M., Garcia Cordova J.M., Villamarin J., Pazmino Zurita J.D., Acosta A. (2026). Obesity and Metabolic Dysfunction-Associated Steatotic Liver Disease (MASLD): A Literature Review on Pathophysiology and Treatment. Diabetes Obes. Metab..

[27] Ghosal S., Ghosal A. (2026). Effects of lifestyle and glucagon-like peptide-1 receptor agonist-based therapies on waist circumference: A systematic review and meta-analysis. Obes. Pillars.

[28] Carli F., Della Pepa G., Sabatini S., Puig A.V., Gastaldelli A. (2024). Lipid metabolism in MASLD and MASH: From mechanism to the clinic. JHEP Rep..

[29] Bansal S.K., Bansal M.B. (2024). Pathogenesis of MASLD and MASH–role of insulin resistance and lipotoxicity. Aliment. Pharmacol. Ther..

[30] Wernberg C.W., Kjer M.F., Grønkjær L.L., Jacobsen B.G., Lauridsen M.M. (2024). Steatotic liver disease is the most important somatic determinant of quality of life in patients with obesity: A cross-sectional study. Liver Int..

[31] Younossi Z.M., Kremer A.E., Swain M.G., Jones D., Bowlus C., Trauner M., Henry L., Gerber L. (2024). Assessment of fatigue and its impact in chronic liver disease. J. Hepatol..

[32] Bril F. (2026). Metabolic liver disease: A summary of major guidelines and identifying opportunities to improve future guidelines. Diabetes Obes. Metab..

[33] Ter Horst K.W., Serlie M.J. (2017). Fructose consumption, lipogenesis, and non-alcoholic fatty liver disease. Nutrients.

[34] Chiavaroli L., Cheung A., Ayoub-Charette S., Ahmed A., Lee D., Au-Yeung F., Qi X., Back S., McGlynn N., Ha V. (2023). Important food sources of fructose-containing sugars and adiposity: A systematic review and meta-analysis of controlled feeding trials. Am. J. Clin. Nutr..

[35] Huber Y., Boyle M., Hallsworth K., Tiniakos D., Straub B.K., Labenz C., Ruckes C., Galle P.R., Romero-Gómez M., Anstee Q.M. (2019). Health-related Quality of Life in Non-alcoholic Fatty Liver Disease Associates with Hepatic Inflammation. Clin. Gastroenterol. Hepatol..

[36] Jamalinia M., Saeian S., Nikkhoo N., Nazerian A., Lankarani K.B. (2025). Sex and gender differences in MASLD: Pathophysiological mechanisms, clinical implications, and future directions. Metab. Target Organ Damage.

[37] Suresh M.G., Mohamed S., Geetha H.S., Prabhu S., Trivedi N., Ng Z.C., Mehta P.D., Brar A.S., Sohal A., Goyal M.K. (2026). Metabolic dysfunction-associated steatotic liver disease and type 2 diabetes: Pathophysiology, diagnosis, and emerging therapeutic strategies. World J. Diabetes.

[38] Cusi K., Abdelmalek M.F., Apovian C.M., Balapattabi K., Bannuru R.R., Barb D., Bardsley J.K., Beverly E.A., Corbin K.D., ElSayed N.A. (2025). Metabolic dysfunction–associated steatotic liver disease (MASLD) in people with diabetes: The need for screening and early intervention. A Consensus Report of the American Diabetes Association. Diabetes Care.

