# Supplementary material for: Dietary Fructose Intake, Metabolic Profile, and Liver Disease-Related Quality of Life in Adults with Obesity and MASLD: A Cross-Sectional Study
Source: Metabolites. 2026 Sep 24;16(10):706. doi: 10.3390/metabo16100706 (PMC13707188; doi:10.3390/metabo16100706)
Supplement: Supplementary file 1 [file metabolites-16-00706-s001.zip › metabolites-4514689-supplementary.pdf]

Supplementary Materials

Supplementary Table S1. Daily nutrient intake by MASLD steatosis grade

Daily nutrient intake was compared across MASLD steatosis grades using the Kruskal–Wallis H test. Most macronutrients, minerals, and vitamins did not differ significantly across steatosis grades (all $p > 0.05$). Two nutrients showed statistically significant overall differences across steatosis grades: Daily water intake ($p = 0.039$), and vitamin A intake ($p = 0.012$). Dunn-Bonferroni post-hoc comparisons did not identify statistically significant pairwise differences for water intake. For vitamin A, intake was significantly higher in Grade 3 than in Grade 2, whereas Grade 1 did not differ signicantly from either group.

Table S1. Daily nutrient intake by MASLD steatosis grade — Kruskal–Wallis test (N = 169).

Nutrient	Grade 1 (n=88) Median (Q1–Q3)	Grade 2 (n=63) Median (Q1–Q3)	Grade 3 (n=18) Median (Q1–Q3)	p
**Macronutrients**				
Energy (kcal)	4291 (3596–5369)	4792 (4123–5472)	5060 (3271–6286)	0.228
Carbohydrate (g)	609 (496–776)	669 (554–751)	675 (515–826)	0.433
Protein (g)	107 (96–135)	125 (99–139)	117 (88–145)	0.416
Fat (g)	153 (113–194)	168 (139–200)	151 (120–215)	0.339
Fructose (g)	105 (89–120)	113 (93–132)	124 (105–138)	0.164
Glucose (g)	107 (90–131)	115 (93–136)	126 (103–141)	0.203
**Dietary fibre**				
Soluble fibre (g)	6.55 (5.47–9.34)	6.49 (5.32–8.81)	6.77 (5.27–8.80)	0.967
Insoluble fibre (g)	20 (14–27)	20 (14–26)	21 (16–28)	0.980
**Hydration**				
Water intake (mL/day)	1500 (1000–2000)	2000 (1000–2500)	2000 (1000–3000)	0.039 *
**Minerals**				
Sodium (mg)	4974 (3664–6086)	5621 (3843–7626)	5531 (2959–7669)	0.313
Magnesium (mg)	535 (433–679)	566 (451–652)	597 (514–633)	0.605
Calcium (mg)	2688 (2251–3283)	2901 (2379–3458)	3024 (1904–3528)	0.354
Potassium (mg)	6165 (4825–7349)	5916 (5094–7178)	6464 (5509–7881)	0.689
**Vitamins**				
Vitamin C (mg)	291 (181–387)	235 (171–395)	369 (167–464)	0.635
Vitamin A (RE)	1022 (706–1588)^{ab}	894 (630–1320)^{a}	1486 (1016–1896)^{b}	0.012 *
Vitamin D (IU)	136 (78–205)	152 (96–221)	175 (88–205)	0.899
Vitamin E (IU)	37 (19–50)	38 (24–54)	38 (27–47)	0.844
Vitamin K2 (mcg)	29 (21–42)	30 (17–40)	27 (18–35)	0.608
Vitamin B6 (mg)	2.10 (1.70–2.57)	2.31 (1.83–2.72)	2.37 (1.75–2.81)	0.460
Vitamin B12 (mcg)	2.92 (1.99–4.52)	3.40 (2.02–4.46)	2.94 (2.01–4.90)	0.794

Data are presented as median (Q1–Q3). Group comparisons were performed using the Kruskal–Wallis H test. Different superscript letters indicate statistically significant differences between groups according to Dunn–Bonferroni post-hoc comparisons ($p < 0.05$). * $p < 0.05$ for the overall Kruskal–Wallis test. Abbreviations: RE, retinol equivalents; MASLD, metabolic dysfunction-associated steatotic liver disease; Q1, 25th percentile; Q3, 75th percentile.

Table S2. Sensitivity analysis after excluding potential energy over-reporters

Outcome	Full sample (n=169), ϱ	p	Plausible energy reporters (n=103), ϱ	p
Waist circumference	0.264	<0.001	0.411	<0.001
HOMA-IR	−0.097	0.209	−0.172	0.082
LDQOL total score	0.122	0.115	0.104	0.294

Values are Spearman's correlation coefficients (ϱ). Fructose intake refers to the questionnaire-derived estimate used as the primary fructose exposure. Energy-reporting plausibility was assessed using the EI/BMR ratio according to the Goldberg/Black approach. BMR was estimated using the sex- and age-specific Schofield equations. For a single-day dietary assessment (d = 1) and an assumed PAL of 1.55, EI/BMR values of 0.87–2.75 were considered plausible. Participants with

EI/BMR >2.75 were classified as potential over-reporters and excluded from the sensitivity analysis. No participant had an EI/BMR <0.87. The PAL value was assumed because individual physical activity levels were not available.

Table S3. Energy-adjusted associations between questionnaire-derived fructose intake and selected outcomes

Outcome	Crude ϱ	p	Energy-adjusted partial ϱ	p
Waist circumference	0.264	<0.001	0.024	0.761
HOMA-IR	−0.097	0.209	−0.102	0.188
LDQOL total score	0.122	0.115	−0.073	0.345
